# Adsorption and Aggregation Behaviors of Oleyl Alcohol-Based Extended Surfactant and Its Mixtures

**DOI:** 10.3390/molecules29112570

**Published:** 2024-05-30

**Authors:** Ping Li, Peiyu Ren, Shuoyu Wang, Jiangshan Wang, Zidan Sun, Jiayi Sun, Weibo Gu

**Affiliations:** High Value Fine Chemicals Research Center, Department of Chemistry and Chemical Engineering, Jinzhong University, Jinzhong 030619, China; destiny02424@126.com (P.R.); wangsy@jzxy.edu.cn (S.W.); wangjs@jzxy.edu.cn (J.W.); sunzd@jzxy.edu.cn (Z.S.); sunjy@jzxy.edu.cn (J.S.); tianruixin_2013@126.com (W.G.)

**Keywords:** oleyl alcohol-based extended surfactant, surface activity, interaction parameter, vesicles, liquid crystal emulsion

## Abstract

An oleyl alcohol-based extended surfactant, sodium oleyl polyethylene oxide-polypropylene oxide sulfate (OE_3_P_3_S), was synthesized and identified using FT-IR and ^1^H NMR. The adsorption and aggregation behaviors of OE_3_P_3_S and its mixture with cationic surfactant alkyltrimethylammoniumbromide (ATAB) were investigated under different molar ratios. The static surface tension analysis indicated that the critical micellization concentration (cmc) and the critical surface tension (γ_cmc_) of OE_3_P_3_S were 0.72 mmol/L, and 36.16 mN/m, respectively. The cmc and γ_cmc_ values of the binary system were much lower than that of the individual component. And the cmc values of OE_3_P_3_S/ATAB = 6:4 mixtures decreased with an increase in the chain length of the cationic surfactant in the binary system. It was found from the dynamic surface tension that there was a slower diffusion rate in the binary system compared to the pure surfactant, and the adsorption processes for OE_3_P_3_S/ATAB = 6:4 were mixed diffusion-kinetic adsorption mechanisms. With a combination of DLS data and TEM measurements, formations of vesicles in OE_3_P_3_S/ATAB = 6:4 solutions appeared to occur at a concentration of 0.05 mmol/L. By studying the formation of liquid crystal structures in an emulsion prepared with OE_3_P_3_S as the surfactant, it was found that the oil-in-water emulsion is birefringent with a Maltese cross texture, and the rheological properties revealed its predominant viscoelastic behavior and shear thinning properties.

## 1. Introduction

Extended surfactants are unique surfactants that contains intermediate polarity groups such as polypropylene oxide (PO) or polypropylene oxide–polyethylene oxide (PO–EO) between the hydrocarbon tail and hydrophilic head group of conventional surfactants [1]. It is reported that extended surfactants possess better interfacial properties and greater salt and temperature tolerances [2,3,4]. Chen and coworkers found that extended surfactants (n-C_c_P_p_S), due to both their rugby-shaped molecular geometry and the dynamic amphipathicity of the PPO spacer, behaved with excellent interfacial and solution properties for household cleaning [5]. Wang et al. proved that a sufficient PO number and low EO number were prerequisites for obtaining ultralow IFT for the extended surfactants, and electrolytes showed little effect on the IFTs of extended surfactants with a “dumbbell” conformation at the interface, because the size of the linear EO group plays a crucial role [2]. Du et al. investigated the effects of PO groups on the wetting properties of C_12_PO_4_S and C_12_PO_11_S solutions on polytetrafluoroethylene (PTFE) surfaces. It was found that the extended surfactant molecules formed a saturated adsorption film at the air–liquid interface but continued to adsorb and form semi-micelles at the solid–liquid interface above the cmc [6]. Considering the abovementioned fine properties, extended surfactants have been investigated in various fields such as enhanced oil recovery, surface modification, and household cleaning [7].

The function of a single surfactant is often not ideal in most cases, and two or more surfactants are usually used in combination to provide a more effective performance and obtain excellent synergies [8]. It has been widely noted that synergism increases with the degree of charge difference; thus, the cationic/anionic surfactant mixtures could gain the highest level of synergism [9]. Accordingly, focused attention on the interfacial properties, structural transformations, and phase behaviors were paid to develop an improved understanding of the synergies between cationic/anionic surfactant mixtures in recent years. For example, experiments by Li and coworkers indicated that the critical micelle concentrations for mixtures of bisquaternary ammonium salt (BQAS) and sodium dodecyl sulfate (SDS) are two orders of magnitude lower than those of either component, and the formation of micelles is an exothermic and entropy increase process [10]. Wang et al. investigated the surface and interfacial properties of mixtures of bisquaternary ammonium salts (16–4–16) and sodium dodecyl benzene sulfonate (SDBS) [9]. Their results showed that the cationic Gemini surfactant 16–4–16 exhibited a strong synergistic effect with SDBS, and the adsorption time, temperature, and liquid–solid ratio had less effect on the IFT of the SDBS/16–4–16 mixture (α_SDBS_ = 0.4) than the cationic surfactant 16–4–16. Zhou et al. studied the effects of sodium thiocyanate (NaSCN) on the mixed decyltriethylammonium bromide and sodium decylsulfonate (C_10_NE–C_10_SO_3_) [11]. The results indicated that the electrostatic attraction between the oppositely charged head groups in the micellar solution of C_10_NE–C_10_SO_3_ was significantly reduced by NaSCN, and the addition of NaSCN could significantly induce a decrease in the aggregate size of C10NE–C_10_SO_3_.

Although a large amount of research has been conducted on the structure and performance of extended surfactants, the properties of mixed systems containing extended surfactants, especially the dynamic surface tension and liquid crystal phase behavior, which play a very important role in practical applications, are still rarely addressed. Herein, an extended surfactant, sodium oleyl polyoxyethylene polyoxypropylene ether sulfate (OE_3_P_3_S), was synthesized, and its structure was confirmed using FT-IR and ^1^H NMR. Then, it was mixed with alkyl trimethyl ammonium bromide (ATAB) at different molar ratios, and the adsorption and aggregation behaviors of OE_3_P_3_S and its mixtures were investigated using static/dynamic surface tension, dynamic light scattering (DLS), and cryo-transmission electron microscopy (cryo-TEM). The properties of liquid crystal emulsions prepared with OE_3_P_3_S as the surfactant were explored using an LxPOL polarizing microscope, small-angle X-ray scattering (SAXS) measurement, and advanced rotational rheometer. This work is expected to provide some basic data and guides for practical applications.

## 2. Results and Discussion

### 2.1. Structural Characterization

**Figure 1:**
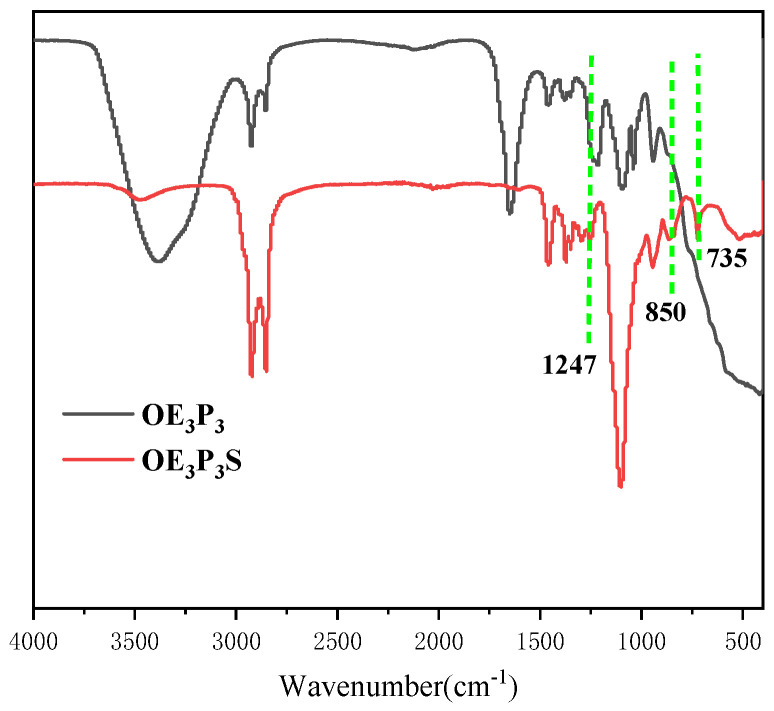
FT-IR spectra of OE_3_P_3_S and OE_3_P_3_.

The FT-IR spectra of OE_3_P_3_S and its raw material oleyl polyoxyethylene polyoxypropylene ether (OE_3_P_3_) are shown in Figure 1. Compared with OE_3_P_3_, E_3_P_3_S shows a symmetrical stretching vibration adsorption peak for S=O bond at 1247 cm^−1^ and stretching vibration absorption peaks for C-O-S at 850 cm^−1^ and 735 cm^−1^, indicating that the sulfation reaction occurred.

**Figure 2:**
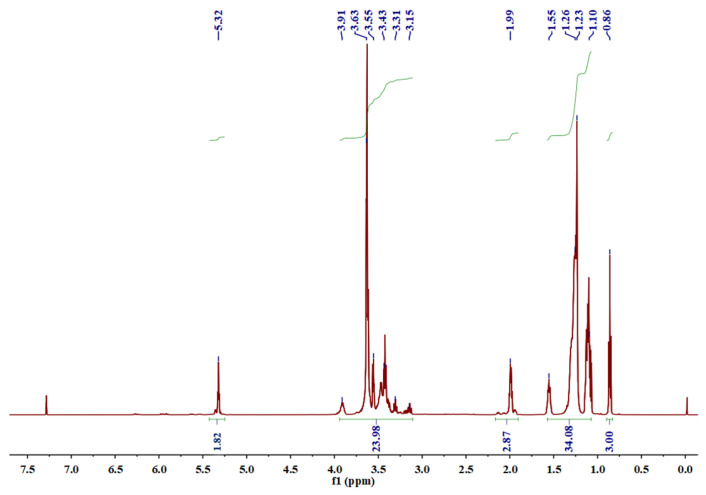
^1^H NMR spectrum of OE_3_P_3_S.

The ^1^H NMR spectrum of OE_3_P_3_S is shown in Figure 2. The results obtained from Figure 2 are listed in Table 1. Based on the relationships between the integral areas of protons and the predicted average adduct numbers corresponding to the protons those are listed in Table 1, the average adduct number for both PO (*m*) and EO (*n*) could be calculated according to the following equations:

S(b):S(a) = (24 + 3*n*):3 = 34.08:3

S(e):S(a) = (2 + 4*m* + 3*n*):3 = 23.98:3

For OE_3_P_3_S, the corresponding values of *m* and *n* were 3.0 and 3.4, respectively.

The analysis of the nuclear magnetic resonance hydrogen spectrum was as follows:

OE_3_P_3_S: ^1^H NMR (400 MHz, CDCl_3_, TMS) δ ppm: 0.86 (t, 3H, -CH_3_), 1.10–1.55 (m, 34H, -CH_2_, -CHCH_3_), 1.99 (m, 4H, -CH_2_CH=CHCH_2_), 3.15–3.91 (m, 24H, -CH_2_-CH_2_-O-CH_2_CH(CH_3_)O-), 5.32 (m, 2H, -CH=CH-).

Combined with the above FT-IR and ^1^HNMR spectrum data, it was demonstrated that OE_3_P_3_S was synthesized successfully.

### 2.2. Krafft Point

The K_T_ value of OE_3_P_3_S is 2 ± 0.5 °C, which is lower than that of sodium oleyl ether sulfate (OE_3_S) (10 ± 2 °C) [12]. It means that the PPO chain make it possible to improve the hydrophobicity of the extended surfactants without sacrificing the water solubility, which is unlike the ordinary hydrocarbon chain on conventional surfactants, because it would also cause an increase in the molecular crystallinity and lead to a sharp increase in the K_T_ [13].

### 2.3. Adsorption Properties

#### 2.3.1. Equilibrium Surface Tension

**Figure 3:**
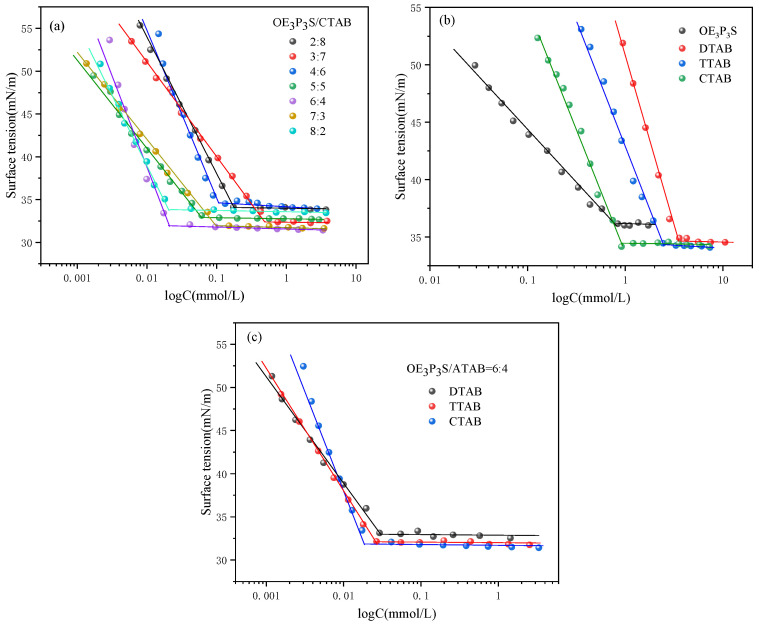
γ-logC curves of aqueous surfactant solutions at 298 K. (**a**) Binary system of OE_3_P_3_S/CTAB with different molar ratios; (**b**)single system; (**c**) binary system of OE_3_P_3_S/ATAB = 6:4.

**Scheme 1:**
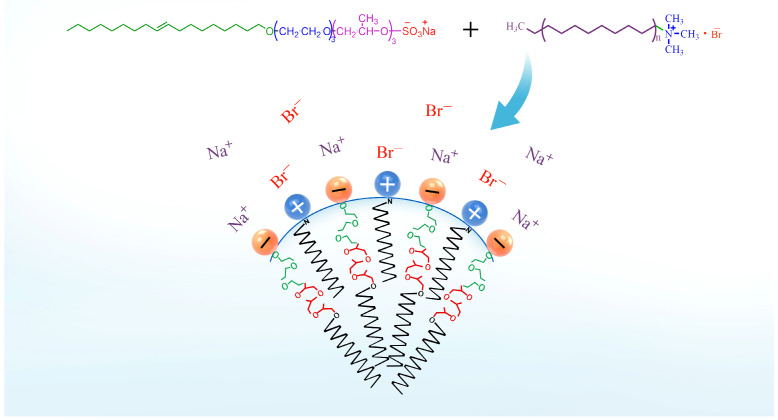
A scheme for the interaction between OE_3_P_3_S and ATAB in the binary system.

The equilibrium surface tension measurement directly revealed the surface activities of the surfactants. The plots of surface tension(γ) versus log molar concentration are shown in Figure 3. The surface tension decreases gradually with concentration increases until it reaches a plateau. The critical surface tension (γ_cmc_) and the corresponding critical micelle concentration (cmc) can be determined from the intersection of two fitting lines, and the equilibrium surface tension performance parameters are listed in Table 2. It is clear from Table 2 that the cmc values of the various mixed sodium oleyl polyoxyethylene polyoxypropylene ether sulfate/cetyltrimethylammonium bromide (OE_3_P_3_S/CTAB) systems are lower compared to those of pure components. This indicates that the binary systems possess better surface tension reduction efficiencies than the single surfactant does. The synergism between the anionic OE_3_P_3_S and cationic CTAB could be ascribed to the stronger electrostatic interaction between oppositely charged head groups along with the hydrophobic interactions between the alkyl chains of the surfactants, leading to easier micelle formation in solution [14]. On comparing different binary systems at the same mixing ratio, the surface tensions of OE_3_P_3_S/ATAB = 6:4 mixtures were also investigated (Figure 3c), since the binary OE_3_P_3_S/CTAB = 6:4 system had the lowest cmc value. The cmc values of sodium oleyl polyoxyethylene polyoxypropylene ether sulfate/dodecayltrimethylaminium bromide (OE_3_P_3_S/DTAB), sodium oleyl polyoxyethylene polyoxypropylene ether sulfate/tetradecyltrimethylammonium bromide (OE_3_P_3_S/TTAB), and OE_3_P_3_S/CTAB (Table 2) were 0.029, 0.026, and 0.018 mmol/L, respectively. It can be seen that the cmc values of OE_3_P_3_S/ATAB = 6:4 mixtures decrease with an increase in the chain length of the cationic surfactant in the mixture. Since the head group remains the same in each of the binary mixtures, a lower cmc is assigned to enhance the hydrophobic interactions between the alkyl tails of the surfactants, leading to the spontaneous formation of mixed micelles. The interaction in the binary system is a physisorption process, and the interaction between OE_3_P_3_S and ATAB is shown in Scheme 1.

The ideal cmc (cmc^ideal^) values for surfactant mixtures in the ideal mixing solutions of a binary system can be calculated using Clint’s equation [15]:

(1) $$\frac{1}{{\mathrm{cmc}}^{\mathrm{ideal}}}=\frac{\alpha_{1}}{{\mathrm{cmc}}_{1}}+\frac{\alpha_{2}}{{\mathrm{cmc}}_{2}}$$

where α_1_ and α_2_ are the molar fractions of surfactants 1 and 2, respectively. The effects of the mixing ratios on the values of the experimental cmc (cmc^exp^) and cmc^ideal^ for OE_3_P_3_S/ATAB mixtures are shown in Figure S1, and the corresponding values are shown in Table 2. As can be seen from Table 2, the values of the cmc^exp^ are lower than those of the cmc^ideal^, indicating the non-ideal mixing behavior in the system. When mixing OE_3_P_3_S and ATAB, there was a synergism caused by the strong electrostatic interaction between oppositely charged head groups, improving the formation of the mixed micelle.

The γ_cmc_ of OE_3_P_3_S/ATAB mixtures can be reduced to about 33 mN/m, which is lower than that of either component. This suggests that the OE_3_P_3_S/CTAB mixtures behave as better surface-active agents in comparison to pure OE_3_P_3_S and ATAB. The enhanced adsorption effectiveness is due to a reduction in the electrostatic repulsions between head groups, resulting in surfactant molecules getting denser and allowing more molecules to get adsorbed at the air/liquid interface per unit area. The γ_cmc_ values of OE_3_P_3_S/DTAB, OE_3_P_3_S/TTAB, and OE_3_P_3_S/CTAB at a mixing ratio of 6:4 are 33.13, 32.75, and 32.13 mN/m, respectively. The very similar γ_cmc_ values are owing to the homologues structure of the cationic surfactant in the mixtures being separated by only a slight difference in the carboxylate chain length.

The surface excess concentration (Γ_max_) and the minimum area per molecule (A_min_) at the air/liquid interface are calculated using the Gibbs adsorption equation [16]:

(2) $$\Gamma_{\max}=-\frac{1}{2.303\mathrm{nRT}}(\frac{\partial \gamma }{\partial \mathrm{lg}c}{)}_{T}$$

(3) $$A_{\min}=\frac{10^{16}}{N_{A}\Gamma_{\max}}$$

where R is the gas constant (8.314 J·mol^−1^·K^−1^), T is the absolute temperature, N_A_ is Avogadro’s constant, and $\frac{\partial \gamma }{\partial \mathrm{lg}c}$ is the slope below the cmc; n = 2 for the pure OE_3_P_3_S and CTAB, and n = 1 for the binary systems. The Γ_max_ and A_min_ obtained are listed in Table 2. As shown in Table 2, the values of Γ_max_ show an opposite trend with respect to A_min_, as expected. The higher Γ_max_ and lower A_min_ of the mixtures are a result of their higher surface activity in the binary systems than the case of the single surfactant, corresponding to the variation in the γ_cmc_. It can be interpreted that the molecules were more densely packed at the air/liquid interface due to the strong electrostatic interactions between oppositely charges and weaker repulsion between the same charges.

The values of the interaction parameters between two components in the binary systems at the adsorbed layers (β_s_) and micelles (β_m_) can be calculated using Equations (4)–(7), respectively.

(4) $$\frac{X_{1s}^{2}\ln(\alpha c_{12}/X_{1s}c_{1})}{{\left(1-X_{1s}\right)}^{2}\ln\left[(1-\alpha )c_{12}/(1-X_{1s})c_{2}\right]}=1$$

(5) $$\beta_{s}=\frac{\ln(\alpha c_{12}/X_{1s}c_{1})}{{\left(1-X_{1s}\right)}^{2}}$$

(6) $$\frac{X_{1m}^{2}\ln(\alpha c_{12m}/X_{1m}c_{1m})}{{\left(1-X_{1m}\right)}^{2}\ln\left[(1-\alpha )c_{12m}/(1-X_{1m})c_{2m}\right]}=1$$

(7) $$\beta_{m}=\frac{\ln(\alpha c_{12m}/X_{1m}c_{1m})}{{\left(1-X_{1m}\right)}^{2}}$$

where X_1s_ and X_1m_ are the molar fractions of component 1 in the mixed adsorbed layers and mixed micelle, respectively. c_1_, c_2_, and c_12_ are the molar concentrations of component 1, component 2, and their mixture under a specified surface tension (γ = 40 mN/m), respectively. c_1m_, c_2m_, and c_12m_ are the critical micelle concentrations of component 1, component 2, and their mixture, respectively. Here, α is the molar fraction of component 1 in the solution system. 

The interaction parameters of OE_3_P_3_S/ATAB mixtures with different mixing ratios at 298 K are listed in Table 3. The X_1s_ and X_1m_ for OE_3_P_3_S/ATAB are found to increase with an increase in the molar fraction of OE_3_P_3_S, which indicates that a greater number of OE_3_P_3_S molecules contribute to the mixed adsorption layers and mixed aggregates. For OE_3_P_3_S/ATAB systems at a mixing ratio of 6:4, the X_1s_ and X_1m_ for OE_3_P_3_S/ATAB can be seen decreasing with an increase in the alkyl chain length of the cationic surfactants in the mixed system, suggesting that a smaller number of ATAB molecules contribute to the mixed adsorption layers and mixed aggregates.

As can be seen in Table 3, both the β_s_ and β_m_ for OE_3_P_3_S/ATAB systems with different mixing ratios are negative, which indicates the attraction effect in the adsorption layer and micelle. The β values are in the range from −5.215 to −16.111 for OE_3_P_3_S/CTAB at various mixing ratios, and the magnitude of the changes is greater than the β values from −5 to −10 for SDS/CTAB mixtures reported by Sohrabi and co-workers [17]. It can be explained that the introduction of an intermediate polarity PPO spacer in the extended surfactant molecule causes a larger attraction effect between the head groups of the surfactants, since the anions and cations are the same for OE_3_P_3_S/CTAB and SDS/CTAB systems. The β_s_ and β_m_ values in Table 3 for OE_3_P_3_S/ATAB at a mixing ratio at 6:4 change from −14.756 to −17.945 and 15.393 to −16.134, respectively. There is not much change in the β values after changing the alkyl chain length of the cationic surfactant in the OE_3_P_3_S/ATAB system, which is similar to the β values ranging from −14.0 to 16.7 for SDBS/[C_n_mim[[Cl] (n = 8, 10, 12) mixtures reported by Shruti and co-workers [14]. A pevious study revealed that β values only expressed the interaction between the head groups of the two surfactants, excluding the interactions between the hydrocarbon chains of surfactants when the chain lengths were different [18].

Thermodynamic parameters can help us further understood the driving force of mixed micelle formation and the interactions between surfactants. Activity coefficients (f_1m_ and f_2m_) in the mixed micelles can be calculated using Equations (8) and (9). Gibbs free energy of micellization and adsorption (ΔG_mic_ and ΔG_ads_), the excess enthalpy of micellization (ΔH_mic_), and the entropy of micellization (ΔS_mic_) can be obtained from Equations (10)–(13), where Π is the surface pressure (γ_0_-γ_cmc_), and C_Π_ refers to the molar concentration of the surfactant in the aqueous phase at a surface pressure Π [19].

(8) $$f_{1m}={e^{\beta_{m}(1-X_{1m})}}^{2}$$

(9) $$f_{2m}={e^{\beta_{m}(X_{1m})}}^{2}$$

(10) $$\Delta G_{mic}=nRT(X_{1m}\ln f_{1m}X_{1m}+X_{2m}\ln f_{2m}X_{2m})$$

(11) $$\Delta G_{\mathrm{ads}}=\Delta G_{mic}-6.022C_{\Pi }A_{\min}\times 10^{-3}$$

(12) $$\Delta H_{mic}=nRT(X_{1m}\ln f_{1m}+X_{2m}\ln f_{2m})$$

(13) $$\Delta S_{\mathrm{mic}}=(\Delta H_{\mathrm{mic}}-\Delta G_{\mathrm{mic}})/T$$

As presented in Table 4, the values of ΔG_mic_ are found to be negative over the whole mixing ratio range for OE_3_P_3_S/ATAB, indicating that the formation of mixed micelles is spontaneous. The more negative ΔG_ads_ suggests that the adsorption process of the mixed surfactant is easier than micellization. The negative ΔH_mic_ and positive ΔS_mic_ indicate that the interactions of binary system are controlled by electrostatic and hydrophobic forces [20]. Moreover, the absolute values of the ratio TΔS_mic_/ΔG_mic_ are lower than 0.5, suggesting that the micellization of OE_3_P_3_S/ATAB mixtures are enthalpically driven processes [20].

#### 2.3.2. Zeta Potential Measurements

**Figure 4:**
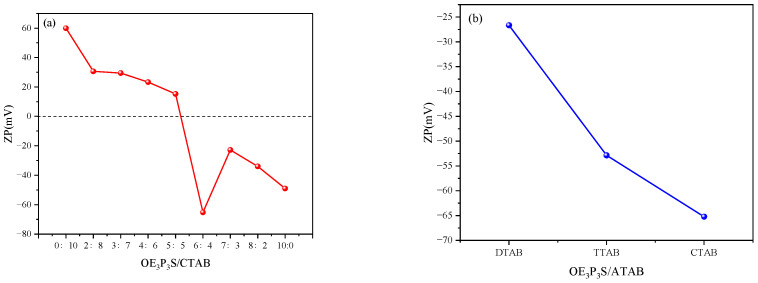
Variation in zeta potential of OE_3_P_3_S/ATAB at 0.01 mmol/L. (**a**) OE_3_P_3_S/CTAB with different molar ratios; (**b**) OE_3_P_3_S/ATAB = 6:4.

The stability of the binary systems was analyzed using zeta potential measurements. The zeta potential is the electrical potential difference between the stationary layer of the colloidal particles and the dispersion medium, which stretches out from the particle surface due to thermal motion of the solvent molecules [21]. Figure 4a shows the variation in the zeta potential of OE_3_P_3_S/CTAB with different molar ratios at 0.01 mol/L. It can be seen that the zeta potential of the CTAB solution is 59.93 mV, reflecting an overall positive charge of the carriers, while the zeta potential value of OE_3_P_3_S is −49 mV. As the content of OE_3_P_3_S increases in the mixture, it begins decreasing due to the partial charge neutralization, as OE_3_P_3_S molecules are negative charge carriers. The charge neutralization indicates the electrostatic interactions between the oppositely charges of the surfactants, which further confirms the results of β_s_ and β_m_. The more OE_3_P_3_S that is added to the mixture, the greater the decrease in the observed surface charge density because of the growth of larger aggregates. The variation in the zeta potential of OE_3_P_3_S/CTAB is consistent with the mixed system of tetradecyltrimethylammonium (TTAB) and imidazolium-based amphiphilic surface-active ionic liquid (BAIL), as reported by Amalendu Pal [22]. The changes in the zeta potential values of the OE_3_P_3_S/ATAB = 6:4 system at 0.01 mmol/L in Figure 4b indicate that the size of the aggregates increases with an increase in the chain length of ATAB. This behavior also supports the results obtained from cryo-TEM.

#### 2.3.3. Dynamic Surface Tension

**Figure 5:**
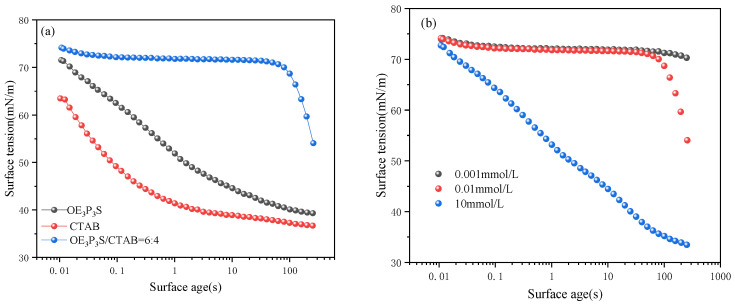
The curve of dynamic surface tension over surface age at 298 K. (**a**) Sodium oleyl polyoxyethylene polyoxypropylene ether sulfate (OE_3_P_3_S), CTAB, and OE_3_P_3_S/CTAB = 6:4 mixture at 0.01mmol/L; (**b**) OE_3_P_3_S/CTAB = 6:4 mixture at 0.001, 0.01, and 10 mmol/L.

Dynamic surface tension (DST) measurements are highly suitable for the study of the diffusion of surfactant molecules from the bulk phase to the interface as well as the adsorption kinetics process [23]. The DST of the binary system of OE_3_P_3_S/CTAB = 6:4 and the related pure surfactant are shown in Figure 5a. In the induction period of the dynamic surface tension curve, the surface tension is constant, or there is a slight almost linear decrease with time [24]. The presence of an induction period could be closely connected to the time required for the adsorption of adequate surfactant molecules at the surface, resulting in appreciable interactions between the adsorbed molecules [25]. It can be clearly seen from Figure 5a that the binary system has a relatively long induction period compared with the single surfactant system, in which the induction period is almost invisible. The ability to reduce the surface tension is due to a combination of various effects, such as the concentration, surfactant type, hydrophobic nature, area per molecule at the interface, and temperature, and in this case, steric hindrance plays a major role [14]. Figure 5b shows the DST of OE_3_P_3_S/CTAB = 6:4 mixtures at 0.001, 0.01, and 10 mmol/L. The induction time decreases with the increase in the binary system concentration. When the concentration is 0.001 mmol/L, the surface tension of the system hardly changes within the surface age of 250 s. This is because the concentration of the system is too low, and the surfactant monomer diffusion rate is slow, making it difficult to achieve effective adsorption at the interface in a short period of time. When the concentration increases to 0.01 mmol/L, the surface tension begins to decrease dramatically at the surface age of 100 s. However, the surface tension decreases significantly from the beginning at a concentration of 10 mmol/L, indicating that there is no induction time under this concentration. Here, the concentration is of critical concern in decreasing the surface tension during the induction stage. There is a rapid decrease in the surface tension after the induction time. The surface tension decrease is determined by the number of surfactant molecules that are in actual contact with the surface [26]. When a new air/liquid interface is created, the concentration of surfactant monomers in the adsorption layer is much less than that in bulk phase, which drives in a transfer of monomers from the bulk solution to the adsorption layer, and the continuous increase in molecules diffusing results in a denser layer and forms the two-dimensional liquid-expanded state. The very fast decrease in the surface tension displays that it is fast adsorbed at the interface, but possibly not the favorable thermodynamic state. And then there is a molecular rearrangement at the interface, for example, a replacement of adsorbed monomers by more surface-active ones [24,27,28]. The overall DST curves shown in Figure 5a reflect that there is a lower dynamic surface activity for the binary system compared to the single surfactant. The binary system seems to be more surface active than the pure surfactant in the equilibrium state, although the opposite was found during the dynamic process. It can be obtained from Figure 5b that the higher the concentrations of the mixture, the more rapid the drop in the surface tension, reflecting the better dynamic surface activity.

**Figure 6:**
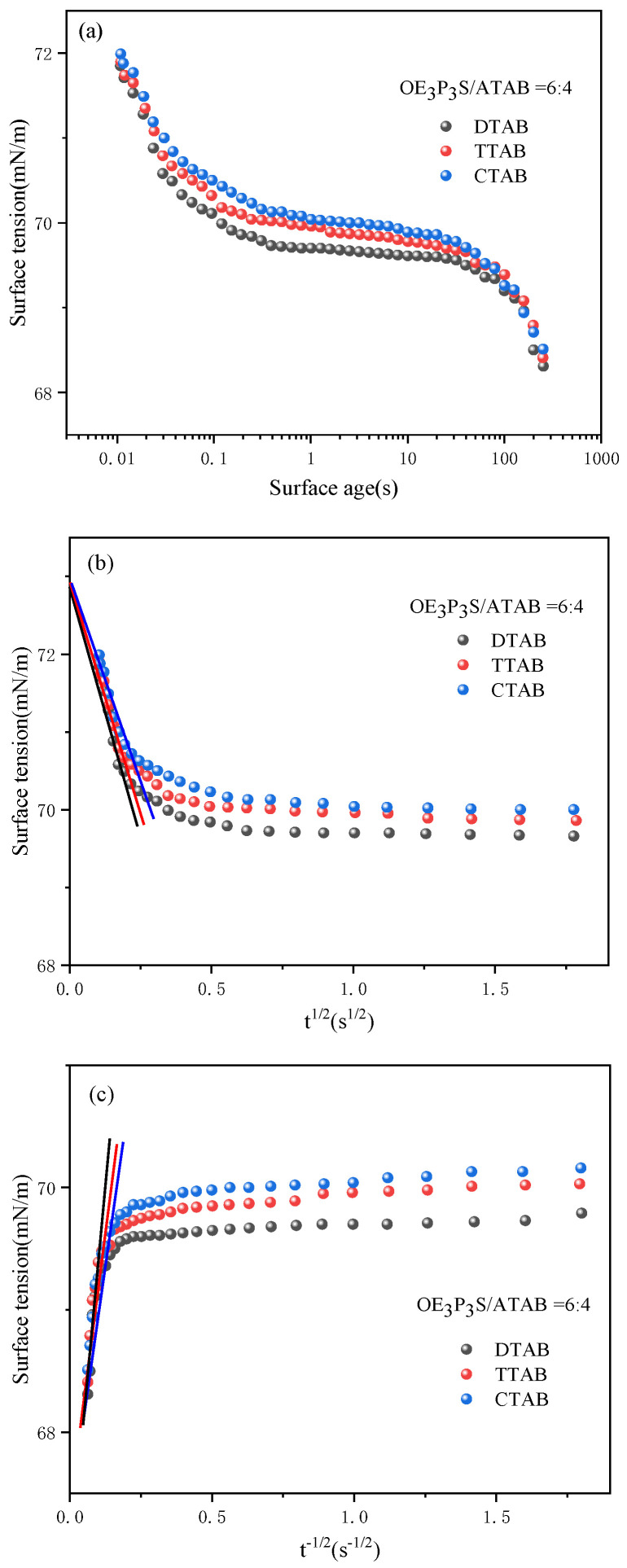
Dynamic surface tension of aqueous OE_3_P_3_S/ATAB = 6:4 mixtures as a function of (**a**) the surface age, (**b**) short time (t^1/2^), and (**c**) long time (t^−1/2^). The OE_3_P_3_S/ATAB = 6:4 mixture concentration was fixed at 0.01 mmol/L. DTAB (black line), TTAB (red line), CTAB (blue line).

The diffusion processes of surfactant molecules in aqueous solutions could be further explored according to the diffusion-controlled adsorption model proposed by Ward–Tordai [29], but due to an incalculable Volterra integral, accounting for back diffusion from the subsurface, the equation cannot be solved. An asymptotic method for solving the Ward–Tordai equation is shown as follows [30]:

(14) $$\text{Short-time}:\;\gamma {\left(t\right)}_{t\to 0}=\gamma_{0}-{2\mathrm{nRTC}}_{0}\sqrt{\frac{\mathrm{Dt}}{\pi }}$$

(15) $$\text{Long-time}:\;\gamma {\left(t\right)}_{t\to \infty }=\gamma_{\mathrm{eq}}+\frac{{\mathrm{nRT}\Gamma }_{\mathrm{eq}}^{2}}{C_{0}}\sqrt{\frac{\pi }{4\mathrm{Dt}}}$$

where C_0_ is the bulk concentration, *π* = 3.142, γ(t) and γ_eq_ represent the surface tension at time and at infinite time, respectively, and Γ_eq_ is the equilibrium surface excess concentration. Equations (14) and (15) enable us to calculate the effective diffusion coefficient from the slope of the DST data plots against t^1/2^ (short time) and t^−1/2^ (long time). Figure 6a shows the effect of the hydrocarbon chain length of the cationic surfactant in the mixture on the rate of surface tension decrease at short adsorption times clearly. This could be interpreted by the fact that long chains probably diffuse slower and adsorb less effectively at the air/liquid interface from the interior of the solution due to the larger steric hindrance [31]. Figure 6b,c display the DST as a function of t^1/2^ and t^−1/2^ for OE_3_P_3_S/ATAB = 6:4 systems at 0.01 mmol/L. In Figure 6b, the plots show a linear behavior on the short time scale, and the values of the intercept are related to the surface tension of the pure solvent. In Figure 6c, the plots also exhibit a linear behavior on the long time scale, and the intercepts are close to the equilibrium surface tension values for the solutions. The values of the effective diffusion coefficients, which were estimated at a short time (D_short_) and long time (D_long_) and obtained from the gradients of the plots in Figure 6b,c, are summarized in Table 5. D_s_ is greater than D_l_ because of the significant concentration difference between the bulk phase and the subsurface in the initial stage of adsorption, which results in the rapid transfer of surfactant molecules from higher to lower concentrations. The D_s_ value gradually decreases with the increase in alkyl chain length of the cationic surfactant in the mixture, which occurs because DTAB has a smaller molecular volume and less spatial potential barrier compared to TTAB and CTAB, thus leading to the faster diffusion of the surfactant molecules from the bulk phase to the subsurface in the initial stage of adsorption. It can also be seen from Table 5 that the changing trend of D_l_ is opposite to that of D_s_. In the later stage of adsorption, the transfer of molecules from the subsurface to the surface layer requires overcoming the surface pressure and adopting an appropriate molecular orientation, and the combined effect of the surface activity and spatial site resistance leads to an increase in D_l_ with the growth in the alkyl chains of the cationic surfactant in the mixture. The ratio of D_l_/D_s_ for OE_3_P_3_S/ATAB = 6:4 was far less than 1, which means that D_l_ is much lower than D_s_, so the adsorption processes for OE_3_P_3_S/ATAB = 6:4 were mixed diffusion-kinetic adsorption mechanisms. Some researchers investigated the dynamics of adsorption of CTAB–silica nanoparticle complexes [31]. The mechanism at CTAB/silica ratios of 2.75 and 5.5 is a mixed kinetic-diffusion controlled mechanism. This means that at these ratios, a kinetic barrier is preventing the complexes from being adsorbed at the interface instantly after they reach the subsurface region. Rong’s group analyzed the dynamic surface tension data for the C_9_pPHCNa/C_10_TABr system using the revised Ward and Tordai equations, and it was shown that the composition of binary surfactants in the bulk solution is an important factor affecting the adsorption kinetics of mixed systems. At α_1_ = 0.33, the minimum electrostatic repulsion between the adsorbed and adsorbing molecules occurs and thus the lowest adsorption barrier and the maximum Da among all the mixed systems [32]. In the case of OE_3_P_3_S/ATAB = 6:4, the OE_3_P_3_S/ATAB system has the largest D_l_ value, indicating that it possesses the lowest adsorption barrier.

### 2.4. Aggregation Behaviors

By preparing samples of the OE_3_P_3_S/CTAB aqueous solution with different mixing ratios at 0.1 mmol/L, it can be seen that the solutions change from transparent to opalescent and then to transparent (see Figure S2). The solubility of the mixture in water varied with the mixing ratio. A better solubility was observed when the mixing ratio was far from 5:5, and phase separation occurred at 4:6. The solution of OE_3_P_3_S/CTAB at 6:4 was bluish and transparent, suggesting the presence of large aggregates. Thus, dynamic light scattering (DLS) and cryo-TEM measurements were performed to further investigate the self-assembly of the solutions.

**Figure 7:**
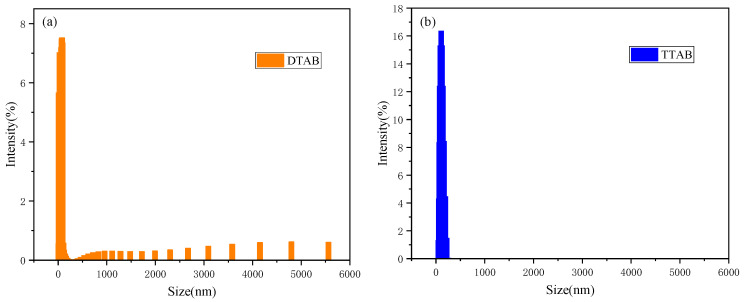
Intensity–size distributions of OE_3_P_3_S/ATAB at a mixing ratio of 6:4 with the concentration of 0.05 mmol/L: (**a**) OE_3_P_3_S/DTAB; (**b**) OE_3_P_3_S/TTAB.

To compare the aggregation behavior of the binary system at the same mixing ratio, the hydrodynamic diameters of the aggregates in OE_3_P_3_S/ATAB solutions at a mixing ratio of 6:4 with concentration of 0.05 mmol/L were determined by means of DLS, and the intensity–size distributions are shown in Figure 7. The hydrodynamic diameters of OE_3_P_3_S/DTAB, OE_3_P_3_S/TTAB, and OE_3_P_3_S/CTAB were concentrated at 78, 106, and 106 nm, respectively. The hydrodynamic diameters were found to be much larger than the small spherical micelles, which have diameters typically around 3–5 nm, suggesting that large aggregates like vesicles might exist in these solutions. It is noteworthy that OE_3_P_3_S/DTAB has the smallest hydrodynamic diameter, which may be due to the shortest chain length of the cationic surfactant participating in the formation of aggregates in the binary system. However, OE_3_P_3_S/TTAB and OE_3_P_3_S/CTAB possess the same size. The reason may be that when the carbon number of the cationic surfactant in a mixed system increases from 12 to 14 and 16, the difference in the chain length has little impact on the formation of aggregates.

**Figure 8:**
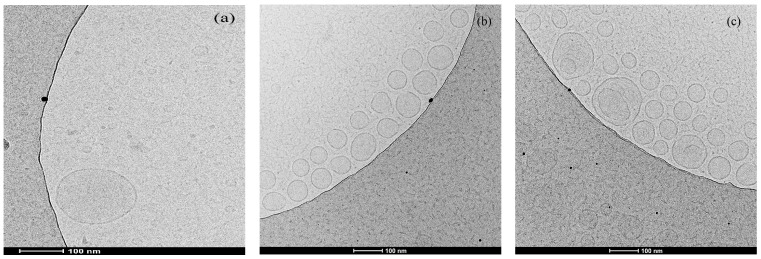
Cryo-TEM images of OE_3_P_3_S/ATAB at a mixing ratio of 6:4 with the concentration of 0.05 mmol/L. (**a**) OE_3_P_3_S/CTAB; (**b**) OE_3_P_3_S/DTAB; (**c**) OE_3_P_3_S/TTAB.

Electron microscope images obtained from cryo-TEM (Figure 8) showed that vesicles formed in OE_3_P_3_S/ATAB solutions at a mixing ratio of 6:4 with a concentration of 0.05 mmol/L. The size distribution of the vesicles for OE_3_P_3_S/DTAB determined using cryo-TEM was in the range between 16 and 416 nm, while the size distributions of the vesicles for OE_3_P_3_S/TTAB and OE_3_P_3_S/CTAB were from 59 to 167 nm and from 74 to 133 nm. As can be seen in Figure 8, OE_3_P_3_S/DTAB possesses the smallest aggregate, while OE_3_P_3_S/TTAB and OE_3_P_3_S/CTAB have larger aggregates with a similar size. This situation is in agreement with that observed in the respective DLS measurement. However, the average size of the systems is much smaller than that determined using DLS. This may be interpreted by the formation of clusters due to the large quantity of vesicles in OE_3_P_3_S/ATAB solutions and the swollen state during the DLS measurement.

Aggregate formed in the OE_3_P_3_S/ATAB solutions could be attributed to the complex formation caused by interactions between the surfactants. Such a complex formation could lead to two results: The first is that the solubility of the complex will be lower than that of the pure surfactant. The greater the degree of combination, the lower the solubility. The second is that hydrophobic groups can participate in the complex through the electrostatic attraction between the oppositely charged ions of surfactants, and this assists hydrophobic aggregation to form self-organized assemblies. The relative sizes of the hydrophilic and hydrophobic areas in the complex will affect the structure of the assemblies. If the hydrophilic area is larger than the hydrophobic area, it will be helpful for the micelle formation; otherwise, it will be beneficial to lamellar structures such as vesicle formation [33]. In the case of our study, a smaller hydrophilic area formed in the complex due to the “neutralization” of the opposite charges of the surfactants in the OE_3_P_3_S/ATAB system at a mixing ratio of 6:4, which is profitable for vesicle formation.

### 2.5. Liquid Crystal Emulsion

**Figure 9:**
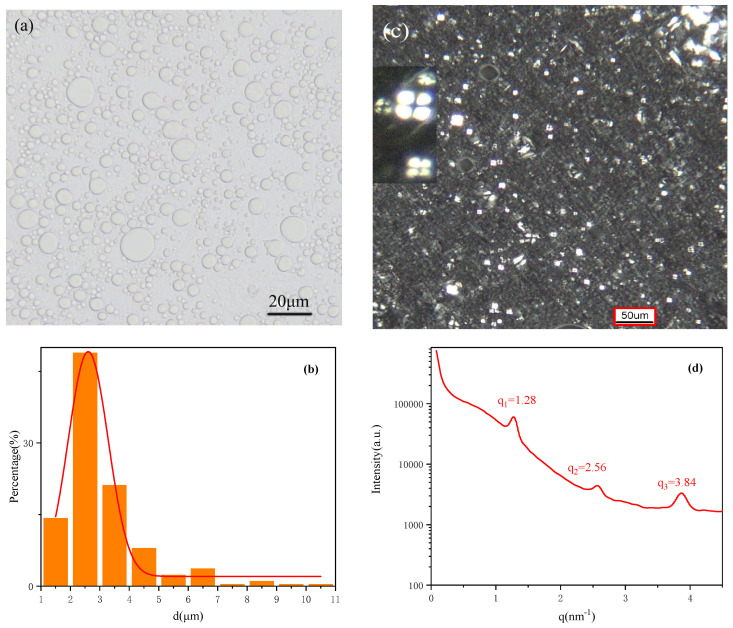
Photographs of emulsions in (**a**) emulsion micrographs, (**b**) diameter distribution, the orange bar is diameter distribution and the red line is diameter distribution fit line, (**c**) structural textures under POM, and (**d**) SAXS.

Liquid crystals possess both liquid and solid properties simultaneously, because they are more organized than liquids but less than solids [34], which makes them appealing for cosmetic use. Among other superiorities, lamellar liquid crystals are similar to the lipidic structure of the stratum corneum, and they have a great skin hydration potential [35,36]. Moreover, liquid crystals can take part in the emulsion stabilization process [37]. Figure 9a displays the droplet image of an emulsion formed by OE_3_P_3_S with cetearyl alcohol, liquid paraffin, and water. The micrograph of the emulsion under a bright field depicts the shape of the acquired oil droplets as being spherical and homogeneous. The results of the particle size distribution analysis using Nano Measurer 1.2 software are complementary to the microscopic observations. As demonstrated in Figure 9b, the size of the emulsion droplets was mainly distributed at 2.61 ± 0.03 µm. The polarized light micrograph in Figure 9c shows that the oil-in-water emulsion is birefringent. Specific textures combined with Maltese crosses indicate the presence of lamellar phases in the system [38,39,40]. The SAXS analysis in Figure 9d shows three obvious scattering peaks, with q values of 1.28, 2.56, and 3.84 nm^−1^, respectively. The scattering factor ratio was q_1_:q_2_:q_3_ = 1:2:3, which confirmed the lamellar liquid crystal phase behavior of the emulsion [41]. The apparent diffraction peak at q_2_ demonstrates the long-range ordering of the liquid crystal structure to make the emulsion less prone to agglomeration and flocculation [40]. The presence of the liquid crystal structure could also typically weaken the van der Waals gravitational force between droplets simultaneously, reducing the aggregation behavior of the droplets and leading to the emulsion being more stable [42].

**Figure 10:**
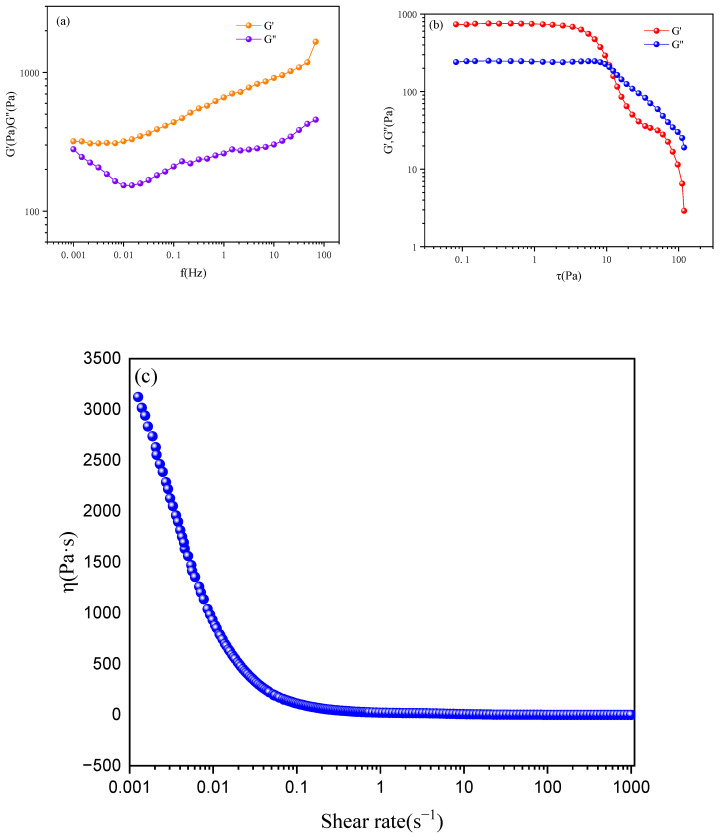
Rheological behavior of emulsion: (**a**) variations in G′ and G″ in relation to shear frequency, (**b**) variation in G′ and G″ in relation to shear stress, and (**c**) viscosity versus shear rate.

The rheological behavior is the physical and mechanical study of material deformation and flow in terms of stress, strain, temperature, and time [43]. For the purpose of understanding the influence of shock on the structure of the layered liquid crystal, an analysis of the dynamic rheological properties was performed. As can be seen in Figure 10a, the elastic modulus G’ is always greater than the viscous modulus G’’ within the tested frequency range, revealing the predominant viscoelastic behavior for the emulsion. A similar phenomenon was previously observed in an emulsion prepared with oleyl ether sulfates (OE_n_S, n = 3, 5, and 7) as the surfactant [12]. The yield stress of the emulsion is shown in Figure 10b, and one can observe that the emulsion began to deform under a low shear force, suggesting a low emulsion strength. Under low shear stress, near 10 Pa, the G’ was greater than the G”, and the modulus decreased, showing that the emulsion mainly existed in the form of unstable elasticity, and the instability of the emulsion was mainly related to a large quantity of water or liquid paraffin oil [43]. As the shear stress continued to increase, both of the two moduli decreased to near 0 Pa, indicating that the emulsion structure had been completely destroyed, which had a smaller value compared to the emulsion prepared with oleyl ether sulfates (OE_n_S, n = 3, 5, and 7) as the surfactant. This meant that our emulsion might have a lower loading capacity. The explanation to this phenomenon could be the branched structure of the PPO groups in the extended surfactant, in our case, influencing the emulsion properties. From the result of the flow test in Figure 10c, one can observe that firstly, the viscosity of the emulsion decreased with the increase in the shear rate and then remained unchanged. This is because under the action of shear force, the force direction of the internal microstructure units of the liquid crystal was parallel to the shear direction, resulting in inter-layer sliding and a decrease in the viscosity. By continuing to increase the shear rate, the microstructure units inside the liquid crystal remain basically consistent or parallel; therefore, it will no longer continue to decline and will maintain a stable value. The good shear thinning properties of the emulsion allowed it to maintain a high viscosity during storage, while allowing it to flow at higher shear rates, making for easy application and spreading [44,45].

## 3. Materials and Methods

### 3.1. Materials

Oleyl polyoxyethylene polyoxypropylene ether (OE_3_P_3_) (industry grade) was purchased from Zhejiang Kaide Chemical Co., Ltd., Hangzhou, Zhejiang, China. Sulfamic acid (SA) and anhydrous ethanol were provided by Shanghai Aladdin Biochemical Technology Co., Ltd., Shanghai, China. N, N-Dimethylformamide (DMF), urea, liquid paraffin, and ATAB were supplied by Tianjin Komio Chemical Reagent Co., Ltd., Tianjin, China. Cetearyl alcohol was purchased from Shanghai Aladdin Biochemical Technology Co., Ltd., Shanghai, China (cosmetic grade). Petroleum ether was offered by Sinopharm Chemical Reagent Co., Ltd., Shanghai, China. All reagents were analytical grade and used without further purification, unless otherwise stated. Ultrapure water with a resistivity of 18.25 MΩ cm was used.

### 3.2. Sample Synthesis and Identification

**Scheme 2:**
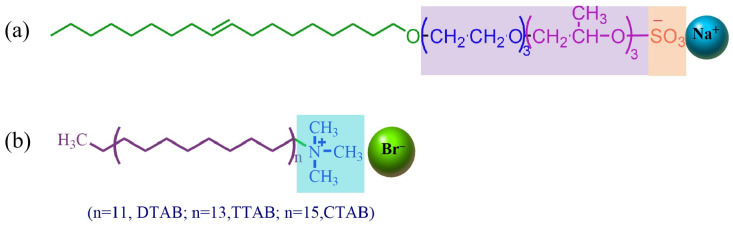
Schematic illustration of the molecular structures of (**a**) OE_3_P_3_S and (**b**)ATAB.

OE_3_P_3_S was synthesized according to Reference [46]. During the reaction process, the molar ratio of OE_3_P_3_ to SA was 1:1.2, SA to urea was 1:0.53, the amount of solvent (DMF) added was 35% of the total reaction material, the reaction temperature was maintained at 100 °C, and the reaction time was 3 h until the end of the sulfation reaction. The reaction mixture was adjusted to pH = 9–10 using 4% NaOH to neutralize the reaction. For detailed purification methods, please refer to [14]. A viscous yellow sample was obtained, and the yield was 92.10%. The sulfation reaction was confirmed using FT-IR (Thermo Scientific Nicolet iS20). The molecular structure of OE_3_P_3_S was confirmed using ^1^H NMR (Bruker 600 M, Billerica, MA, USA) in CDCl_3_. The general molecular formulas of OE_3_P_3_S and ATAB are illustrated in Scheme 2.

### 3.3. Krafft Point (K_T_)

OE_3_P_3_S solutions were prepared at 1 wt% and placed in a refrigerator at −20 °C for 24 h. The K_T_ was determined by heating the above solution at a rate of 1 °C/min, observing the change from turbid to clarified solution. If the solution remained clear even below −4 °C, it would be marked as K_T_ < 0 °C.

### 3.4. Measurements

The equilibrium surface tension was determined through a continuous method by using Krüss K12 (Krüss, Hamburg, Germany) with a platinum ring at 25.0 ± 0.1 °C. The zeta potential test was performed on a Zetasizer Nano ZS (Malvern Panalytical, Malvern, UK). The dynamic surface tension of the solutions was measured using the bubble pressure method with a BP-100 dynamic surface tensiometer (Krüss, Hamburg, Germany) at 25.0 ± 0.1 °C. All the solutions were aged for at least 24 h before the surface tension measurements were carried out. The morphology and size of the aggregates were characterized using cryo-TEM, operated at 120 kV (JEOL JEM-1400 TEM, JEOL, Tokyo, Japan), and the dynamic light scattering technique (Zetasizer Nano ZS, Malvern Panalytical, Malvern, UK). The liquid crystal phase structure was analyzed using the LxPOL polarizing microscope (LaboAmerica, Fremont, CA, USA) and small-angle X-ray scattering (SAXS) measurement using a tweezer-filtered Cu Kα radiation Anton-Paar SAX Sess mc^2^ system at 50 kV and 40 mA (Xenocs, Grenoble, France). The morphology of the emulsion can be observed using the LxPOL POM under a bright field, and the size of emulsion droplets was analyzed using Nano Measurer 1.2 software. The rheological behavior of the emulsion was observed using the advanced rotational rheometer MCR 302 (Anton Paar, Graz, Austria).

### 3.5. Preparation of Emulsion

The emulsion was a multi-mixed system involving water, liquid paraffin, and emulsifier [44], where the emulsifier was made up of cetearyl alcohol and the surfactant OE_3_P_3_S. The emulsion was prepared by heating 10% of the emulsifier, in which the mass ratio of cetearyl alcohol to OE_3_P_3_S was 6:4, 20% liquid paraffin, and 70% ultrapure water up to 75 °C under mechanical stirring at 10,000 rpm for 5 min. The above mixture was cooled to room temperature, and a creamy emulsion was obtained.

The microscope slides were prepared by putting a certain amount of the emulsion on the slide and pressing gently with a coverslip to make it as thin as possible. And then the slide was observed with a polarizing microscope under a bright field and polarized light [46]. The bright field image was used to analyze the droplet size, and the polarized light image was employed to observe the oil streaks or Maltese crosses.

## 4. Conclusions

The extended surfactant OE_3_P_3_S was synthesized and compounded with alkyl trimethyl ammonium bromide (ATAB) to investigate the properties of the single and mixed surfactant systems. The K_T_ value of OE_3_P_3_S is 2 ± 0.5 °C, indicating that the PPO chain makes it possible to improve the hydrophobicity of the extended surfactant without sacrificing water solubility. The adsorption properties indicate that the surfactant mixtures exhibit strong synergistic effects in the reduction of surface tension and the formation of micelles. The cmc values of OE_3_P_3_S/ATAB = 6:4 mixtures decrease with an increase in the chain length of the cationic surfactant in the system. Moreover, there was a slower diffusion rate in the binary system compared to the pure surfactant, and the adsorption processes for OE_3_P_3_S/ATAB = 6:4 were mixed diffusion-kinetic adsorption mechanisms. The aggregation behavior shows that it can self-assemble into vesicles in the binary system of OE_3_P_3_S/ATAB at a mixing ratio of 6:4. The properties of the liquid crystal emulsion prepared with OE_3_P_3_S as the surfactant reveals that the emulsion had a predominant viscoelastic behavior and shear thinning properties.

This work provides a substantial understanding of the surface properties and aggregation behavior for an oleyl alcohol-based extended surfactant and its mixtures, and it is expected to be useful in designing formulations for practical applications.

## Figures and Tables

**Table 1:** The resolution of the ^1^H NMR spectrum of OE_3_P_3_S.

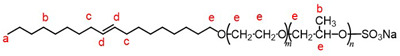			
	*δ*_H_ (ppm)	Integral Areas of Protons	Predicted Proton Numbers
a	0.81~1.00	3.00	3
b	1.00~1.55	36.08	24 + 3*n*
e	3.10~3.91	23.98	2 + 4*m* + 3*n*

**Table 2:** Parameters of surface properties for sodium oleyl polyoxyethylene polyoxypropylene ether sulfate (OE_3_P_3_S) and cetyltrimethylammonium bromide (CTAB), dodecayltrimethylaminium bromide (DTAB), or tetradecyltrimethylammonium bromide (TTAB) mixtures with different mixing ratios at 298 K.

Systems		cmc^exp^ (mmol/L)	cmc^ideal^ (mmol/L)	γ_cmc_ (mN/m)	A_min_ (nm^2^/molecule)	Γ_max_ (μmol/m^2^)
OE_3_P_3_S/CTAB	0:10	0.94	0.94	34.89	107.13	1.55
	2:8	0.18	1.06	33.06	62.30	2.67
	3:7	0.50	1.00	33.02	91.76	1.81
	4:6	0.11	0.95	33.40	41.62	3.99
	5:5	0.065	0.90	33.03	99.62	1.67
	6:4	0.016	0.86	32.13	35.21	4.72
	7:3	0.088	0.82	32.19	91.11	1.82
	8:2	0.017	0.78	33.93	56.02	2.97
	10:0	0.72	0.72	36.16	203.26	0.82
OE_3_P_3_S/DTAB	6:4	0.029	0.068	33.13	78.95	2.10
OE_3_P_3_S/TTAB	6:4	0.026	0.062	32.75	67.76	2.45

**Table 3:** Interaction parameters of OE_3_P_3_S/ATAB mixtures with different mixing ratios at 298 K.

Systems		X_1s_	X_1m_	β_s_	β_m_
OE_3_P_3_S/CTAB	2:8	0.452	0.453	−7.446	−7.378
	3:7	0.475	0.465	−5.215	−2.805
	4:6	0.504	0.505	−7.628	−8.617
	5:5	0.514	0.518	−13.368	−10.505
	6:4	0.524	0.523	−14.756	−16.134
	7:3	0.546	0.558	−12.470	−9.619
	8:2	0.552	0.551	−16.111	−16.706
OE_3_P_3_S/DTAB	6:4	0.556	0.550	−17.945	−15.403
OE_3_P_3_S/TTAB	6:4	0.545	0.543	−17.285	−15.393

**Table 4:** Thermodynamic parameters of OE_3_P_3_S/ATAB mixtures with different mixing ratios at 298 K.

Systems		ΔG_mic_ (KJ/mol)	ΔG_ads_ (KJ/mol)	ΔH_mic_ (KJ/mol)	TΔS_mic_ (KJ/mol)	|TΔS_mic_/ΔG_mic_|
OE_3_P_3_S/CTAB	2:8	−6.24	−20.47	−4.53	1.71	0.27
	3:7	−3.44	−24.43	−1.73	1.71	0.49
	4:6	−7.05	−16.48	−5.34	1.72	0.24
	5:5	−8.21	−30.99	−6.50	1.72	0.21
	6:4	−11.69	−19.93	−9.97	1.71	0.15
	7:3	−7.58	−28.87	−5.88	1.70	0.22
	8:2	−11.94	−24.45	−10.24	1.70	0.14
OE_3_P_3_S/DTAB	6:4	−11.15	−29.16	−9.45	1.71	0.15
OE_3_P_3_S/TTAB	6:4	−11.17	−26.78	−9.46	1.71	0.15

**Table 5:** Effective diffusion coefficients of OE_3_P_3_S/ATAB = 6:4 mixtures at 298 K.

Systems	D_s_ (m^2^/s)	D_l_ (m^2^/s)	D_l_/D_s_
OE_3_P_3_S/DTAB	4.35 × 10^−11^	1.24 × 10^−14^	0.00029
OE_3_P_3_S/TTAB	3.21 × 10^−11^	1.66 × 10^−14^	0.00052
OE_3_P_3_S/CTAB	2.60 × 10^−11^	1.95 × 10^−13^	0.0075

## Data Availability

Data are contained within the article.

## Supplementary Materials

The following supporting information can be downloaded at: https://www.mdpi.com/article/10.3390/molecules29112570/s1, Figure S1: Variations in the cmc^exp^ and cmc^ideal^ for OE_3_P_3_S/CTAB at different mixing ratios; Figure S2: Samples of OE_3_P_3_S/CTAB at different mixing ratios.
