# Supplementary material for: Adsorption and Aggregation Behaviors of Oleyl Alcohol-Based Extended Surfactant and Its Mixtures
Source: Molecules. 2024 May 30;29(11):2570. doi: 10.3390/molecules29112570 (PMC11173569; doi:10.3390/molecules29112570)
Supplement: Supplementary file 1 [file molecules-29-02570-s001.zip › molecules-2994527-supplementary.pdf]

Supporting information

Adsorption and Aggregation Behaviors of Oleyl Alcohol-Based Extended Surfactant and Its Mixtures

Ping Li *, Peiyu Ren, Shuoyu Wang, Jiangshan Wang, Zidan Sun, Jiayi Sun and Weibo Gu

High Value Fine Chemicals Research Center, Department of Chemistry and Chemical Engineering, Jinzhong University, Jinzhong 030619, China; destiny02424@126.com (P.R.); wangsy@jzxy.edu.cn (S.W.); wangjs@jzxy.edu.cn (J.W.); sunzd@jzxy.edu.cn (Z.S.); sunjy@jzxy.edu.cn (J.S.); tianruixin_2013@126.com (W.G.)

* Correspondence: yipingli_@126.com; Tel.: +86-13485350814

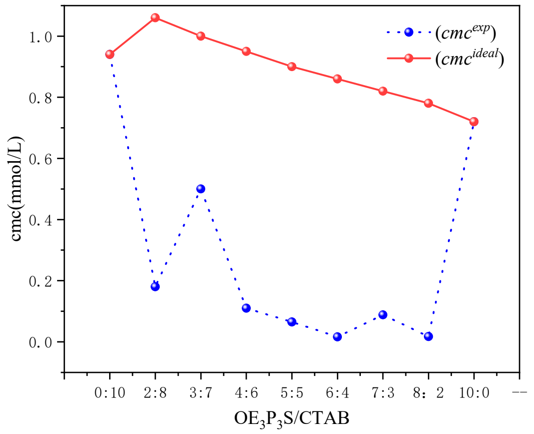

Figure S1. Variation of the cmc^{exp} and cmc^{ideal} for OE_3P_3S/CTAB at different mixing ratios.

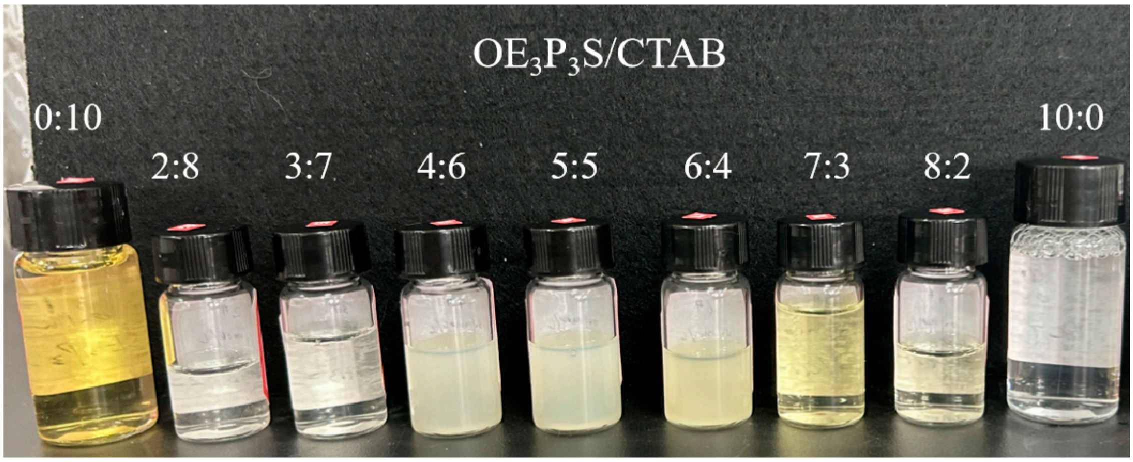

Figure S2. Samples of OE_3P_3S/CTAB at different mixing ratios.
